# Knockdown of GPSM1 Inhibits the Proliferation and Promotes the Apoptosis of B-Cell Acute Lymphoblastic Leukemia Cells by Suppressing the ADCY6-RAPGEF3-JNK Signaling Pathway

**DOI:** 10.3389/pore.2021.643376

**Published:** 2021-04-07

**Authors:** Ye Zhang, Bo Zhou, Jingjing Sun, Qun He, Yujie Zhao

**Affiliations:** ^1^ Key Laboratory of Cell Biology of Ministry of Public Health, and Key Laboratory of Medical Cell Biology of Ministry of Education, China Medical University, Shenyang, China; ^2^ Department of Bioinformatics, School of Life Sciences, China Medical University, Shenyang, China

**Keywords:** B-cell acute lymphoblastic leukemia, GPSM1, ADCY6, RAPGEF3, JNK

## Abstract

B-cell acute lymphoblastic leukemia (B-ALL) is the common type of blood cancer. Although the remission rate has increased, the current treatment options for B-ALL are usually related to adverse reactions and recurrence, so it is necessary to find other treatment options. G protein signaling modulator 1 (GPSM1) is one of several factors that affect the basic activity of the G protein signaling system, but its role in B-ALL has not yet been clarified. In this study, we analyzed the expression of GPSM1 in the Oncomine database and found that the GPSM1 levels were higher in B-ALL cells than in peripheral blood mononuclear cells (PBMCs). Analyses of the Gene Expression Profiling Interactive Analysis (GEPIA) demonstrated that patients with high GPSM1 levels had shorter survival times than those with low levels. Additionally, gene set enrichment analysis (GSEA) suggested that GPSM1 was positively correlated with proliferation, G protein-coupled receptor (GPCR) ligand binding, Gαs signaling and calcium signaling pathways. In further experiments, GPSM1 was found to be highly expressed in Acute lymphoblastic leukemia (ALL) cell lines, and downregulation of GPSM1 inhibited proliferation and promoted cell cycle arrest and apoptosis in BALL-1 and Reh cells. Moreover, knockdown of GPSM1 suppressed ADCY6 and RAPGEF3 expression in BALL-1 and Reh cells. Furthermore, we reported that GPSM1 regulated JNK expression via ADCY6-RAPGEF3. The present study demonstrates that GPSM1 promotes tumor growth in BALL-1 and Reh cells by modulating ADCY6-RAPGEF3-JNK signaling.

## Introduction

Acute lymphoblastic leukemia (ALL) involves the malignant transformation and proliferation of lymphoid progenitor cells in the bone marrow, blood and extramedullary sites (1). Many predisposing risk factors could be associated with ALL, such as exposure to radiation, or chemicals, genetic characteristics or viral infections (2). G protein-coupled receptors (GPCRs) are the largest family of targets for approved drugs (3). GPCRs and their downstream components play an important role in cancer initiation and progression, and they can influence aberrant cell growth and survival (4). Several studies have shown that GPCR dysregulation was found in B-cell acute lymphoblastic leukemia (5), acute myeloid leukemia (6), chronic lymphocytic leukemia (7, 8) and intestinal T-cell lymphoma (9). However, the mechanism by which GPCRs and their downstream effectors regulate the development of ALL remains to be discovered.

GPSM1, also referred to as activator of G protein signaling 3 (AGS3), acts as a receptor-independent activator of G protein signaling. It is a guanine nucleotide dissociation inhibitor, that inhibits the dissociation of guanine diphosphate (GDP) from Gα subunits and competitively prevents Gβγ subunits from coupling with Gα subunits (10–12). In addition to its regulatory role in GPCR signaling, GPSM1 has been shown to mediate several cellular functions, including asymmetric cellular division (13), autophagy (12), intracellular pathogen clearance (14), protein trafficking (15), behavioral changes to addiction (16), polycystic kidney disease (17) and chemotaxis (18).

However, to our knowledge, no studies have investigated the effects of GPSM1 in leukemia. This study aimed to investigate the role and regulatory mechanism of GPSM1 in B-ALL. First, the expression, prognostic value and functional mechanism of GPSM1 in leukemia were analyzed by utilizing certain bioinformatics methods. Subsequently, we investigated the expression of GPSM1 in ALL cell lines and indicating that GPSM1 plays a significant role in ALL. We demonstrated that downregulation of GPSM1 in BALL-1 and Reh cells was associated with a reduction in cell proliferation, inhibition of cell cycle progression and enhancement of apoptosis through inhibition of the ADCY6-RAPGEF3-JNK pathway. These results identified GPSM1 as a potential target for the treatment of B-ALL.

## Materials and Methods

### Database Analysis of GPSM1

The mRNA expression of GPSM1 in B-ALL was analyzed within the Oncomine (www.oncomine.org) database (19). Survival prediction was performed using the Gene Expression Profiling Interactive Analysis (GEPIA) (20) (http://gepia.cancer-pku.cn/). A total of 173 LAML and 70 normal samples with gene expression data (TPM) and clinical information were collected from TCGA (https://portal.gdc.cancer.gov/) and GTEx (https://gtexportal.org/). ROC (receiver operating characteristic) analysis was then performed by pROC package (21) to assess the effectiveness of the transcriptional expression of GPSM1 to discriminate acute myeloid leukemia from healthy samples. The computed area under the curve (AUC) value ranging from 0.5 to 1.0 indicates the discrimination ability from 50 to 100%. Gene set enrichment analysis (GSEA) was performed using GSEA 4.1.0 software (http://www.broadinstitute.org/gsea/). The GSE87070 dataset was analyzed to determine the signaling pathways related to GPSM1 expression in B-cell precursor ALL (BCP-ALL). GSEA of GPSM1 was performed using the Pearson correlation coefficient with 1,000 permutations against collection C2 (curated gene set), which is publicly available at MsigDB (http://www.broad.mit.edu/gsea/msigdb/index.jsp). All other parameters were set to the default values. *p* < 0.05 was chosen as the significance cutoff criterion. The leading-edge genes were mapped by means of the Kyoto Encyclopedia of Genes and Genomes (KEGG) pathway database using KEGG Mapper (https://www.genome.jp/kegg/mapper.html). UALCAN (http://ualcan.path.uab.edu/) was used for gene expression correlation analysis.

### Antibodies and Reagents

Anti‐GPSM1 (sc-271721, 1:1,000 dilution) was purchased from Santa Cruz (Santa Cruz Biotechnology, Santa Cruz, CA). Anti‐GAPDH (60004-1-Ig, 1:10,000 dilution) and anti‐ADCY6 (14616-1-AP, 1:1,000 dilution) were purchased from Proteintech (Wuhan, China). Anti‐RAPGEF3 (4,155, 1:1,000 dilution) and anti-JNK (9,252, 1:1,500 dilution) were purchased from Cell Signaling Technology (Cell Signaling Technology Inc., MA, United States). The RAPGEF3 inhibitor ESI-09 was purchased from Selleck Chemicals (Selleck, United States). ESI-09 concentrations used to test dose-dependent inhibition were at 0, 10, 20, 40 μM for 24 h. To examine the time-dependency, cells were incubated with 20 μM ESI-09 for 0, 12, 24 36 h.

### Cell Culture

A human B lymphoblast cell line (HMy2.CIR) was purchased from Procell Life Science and Technology Co., Ltd (Wuhan, China). A human B-ALL cell line (BALL-1) was obtained from the Chinese Academy of Sciences (Kunming, China). A human T-cell lymphoblastic leukemia cell line (Jurkat) was obtained from Procell Life Science and Technology Co., Ltd. A human lymphoblastic leukemia cell line (Reh) was obtained from Shanghai Genechem Co., Ltd (Shanghai, China). HMy2.CIR cells were cultured in Iscove’s modified Dulbecco’s medium (IMDM; Gibco, Grand Island, NY, United States) containing 10% fetal bovine serum (FBS; Gibco), 100 U/ml penicillin, and 100 μg/ml streptomycin (Gibco). BALL-1, Jurkat and Reh cells were cultured in RPMI medium 1,640 (Gibco) supplemented with 10% FBS and antibiotics (100 U/ml penicillin and 100 μg/ml streptomycin). All cells were cultured at 37°C in an incubator with a 5% CO_2_ atmosphere. Logarithmically growing cells were used in all experiments.

### Adenovirus-Meditated RNA Interference

The knockdown study was performed using adenovirus-mediated RNA interference. Adenoviral transduction particles were purchased from HanHeng Trading Co. Ltd. (Shanghai, China). GPSM1 shRNA (sh-GPSM1) and a scrambled shRNA (sh-Con) sequences are described in Table1. Infection with the adenovirus was executed in accordance with manufacturer’s product protocol. Briefly, cells were plated in 6-well plates at 1 × 10^6^ cells/well. BALL-1 and Reh cells were then infected with 100 MOI (multiplicity of infection)/ml prepared virus in 1 ml of media for 48 h. The adenovirus was subsequently washed off with PBS, and the cells underwent the indicated treatments. The knockdown effect was evaluated at the RNA level by real-time PCR and at the protein level by Western blot analyses as described in the following sections.

**TABLE 1:** GPSM1-shRNA sequences used for insertion in adenoviruses.

Gene targeted	Strand	shRNA sequence
sh-GPSM1	Top strand	AAT​TCG​TGG​CGC​CTA​CAA​GCC​AGT​TCT​TCT​TTT​CAA​GAG​AAA​GAA​GAA​CTG​GCT​TGT​AGG​CGC​CAT​TTT​TTG
	Bottom strand	GAT​CCA​AAA​AAT​GGC​GCC​TAC​AAG​CCA​GTT​CTT​CTT​TCT​CTT​GAA​AAG​AAG​AAC​TGG​CTT​GTA​GGC​GCC​ACG
sh-Con	Top strand	GAT​CCG​TTC​TCC​GAA​CGT​GTC​ACG​TAA​TTC​AAG​AGA​TTA​CGT​GAC​ACG​TTC​GGA​GAA​TTT​TTT​C
	Bottom strand	AAT​TGA​AAA​AAT​TCT​CCG​AAC​GTG​TCA​CGT​AAT​CTC​TTG​AAT​TAC​GTG​ACA​CGT​TCG​GAG​AAC​G

### Quantitative Real-Time PCR (RT-qPCR)

Total RNA was obtained from cells using TRIzol reagent (Life Technologies, Waltham, MA, United States). A total of 1 μg RNA from each sample was reverse-transcribed using the PrimeScript™RT reagent kit with gDNA Eraser (TakaRa, Japan) and amplified using SYBR *Premix Ex Taq* ^TM^ Ⅱ (TakaRa) on a StepOne™ Real-Time PCR System (Life Technologies). The data were calculated using the following equation: relative mRNA expression = 2^−ΔΔCt^, where ΔΔCt = (Ct sample − Ct control) treatment − (Ct sample − Ct control) normal. GAPDH was used as internal control. All primers were designed and purchased from Sangon Biotech (Shanghai, China) (Table 2).

**TABLE 2:** Primer sequences for RT-qPCR.

Gene name	Forward primer	Reverse primer
GPSM1	ATG​GAG​ACA​GCC​ACC​ATT​CA	CCT​GGT​ACT​TCC​TGC​TCC​TC
GADPH	CAG​GAG​GCA​TTG​CTG​ATG​AT	GAAGGCTGGGGCTCATTT
ADCY6	AGA​CAT​TGA​GGG​CTT​CAC​CA	GGG​CAA​AGA​GCT​CAT​TCA​GG
RAPGEF3	AGC​GAG​AAT​TAG​CGG​CTG​TT	GGG​TCA​CCA​CGT​TGA​CAG​AT
JNK	ACT​ACA​GAG​CAC​CCG​AGG​TC	TCC​CAT​GAT​GCA​CCC​AAC​TG

### Western Blot Analysis

Total protein was extracted by lysing samples in RIPA buffer with 1% PMSF, and the protein levels were quantified by a protein quantitative analysis kit (KeyGEN, China). Cell lysates were diluted at a ratio of 1:5 with protein loading buffer (6×) and heated at 100°C for 5 min. Thirty micrograms of each protein sample was separated on 10% SDS-PAGE gels at 100 V for 2 h and then transferred to polyvinylidene fluoride (PVDF) membranes (Millipore, United States) for 120 min at 100 mA. After blocking in 5% nonfat dried milk in TBST for at least 1 h, the membranes were incubated with the primary antibodies listed under Reagents in TBST overnight at 4°C. After washing three times with TBST, the bands were then incubated with HRP-linked secondary antibody anti-rabbit IgG or anti-mouse IgG (Cell Signaling Technology). The results were visualized via the SuperSignal West Pico Chemiluminescent Substrate kit (Thermo, United States). Densitometry of the immunoreactive bands was performed with Tanon Image software. GAPDH was regarded as an internal control.

### Cell Viability Assay

The Cell Counting Kit-8 (CCK-8, KeyGEN, China) assay was used to assess cell viability. In each group, BALL-1 and Reh cells (100 μl) were seeded in a 96-well plate at a density of 1 × 10^5^ cells/ml. Cell viability was determined at 0 h and every 24 h over the following four days. Ten microliters of CCK-8 were added to each well and cultured for 2 h. The optical density (OD) value at a 450 nm wavelength was determined by a microplate reader (BioTek Instruments Inc., Winooski, VT, United States). All experiments were performed three times, and each experiment contained three replicates.

### Cell Cycle Analysis

Cells were fixed in 70% ice-cold ethanol overnight. Then, the cells were washed with PBS and stained with 0.5 ml PI/RNase Staining Buffer (BD Biosciences, CA, United States) at room temperature for 15 min. Cell-cycle phase distributions were determined on a flow cytometry (Calibur, BD Biosciences) and analyzed by the ModFitLT V3.0 program (BD Biosciences). The percentage of cells in G1, S, and G2 phase were counted and compared. Tests were performed three times for each sample.

### Apoptosis Analysis

Apoptosis was measured by annexin V-PE/7-AAD double-staining (Annexin V-PE/7-AAD Apoptosis Detection Kit, BD Biosciences). In each group, BALL-1 and Reh cells (1 × 10^6^ cells/tube) were collected and washed with PBS, and then resuspended in 100 μl 1× binding buffer. Then, the cell suspension was incubated with 5 μl annexin V-PE and 5 μl 7-AAD at room temperature for 15 min. After this incubation, 400 μl 1× binding buffer was added to the cells before analysis with a LSRFortessa flow cytometer (BD Biosciences). FACSDiva software (version 6.2, BD Biosciences) was used for data analysis. The early apoptosis and late apoptosis rates are indicated as the percentage of annexin V-PE^+^/7-AAD^−^ or annexin V-PE^+^/7-AAD^+^ cells, respectively.

### Statistical Analysis

All data were analyzed using GraphPad Prism 7.0 software (GraphPad Software, San Diego, CA, United States) and expressed as the mean ± SD of at least three experiments. Comparisons between two groups were performed using Student’s *t* test, and comparisons among multiple groups were performed using one-way ANOVA with post-hoc intergroup comparisons using Tukey’s test. Differences with *p* < 0.05 were considered statistically significant.

## Results

### GPSM1 Was Highly Expressed in B-ALL

**FIGURE 1:**
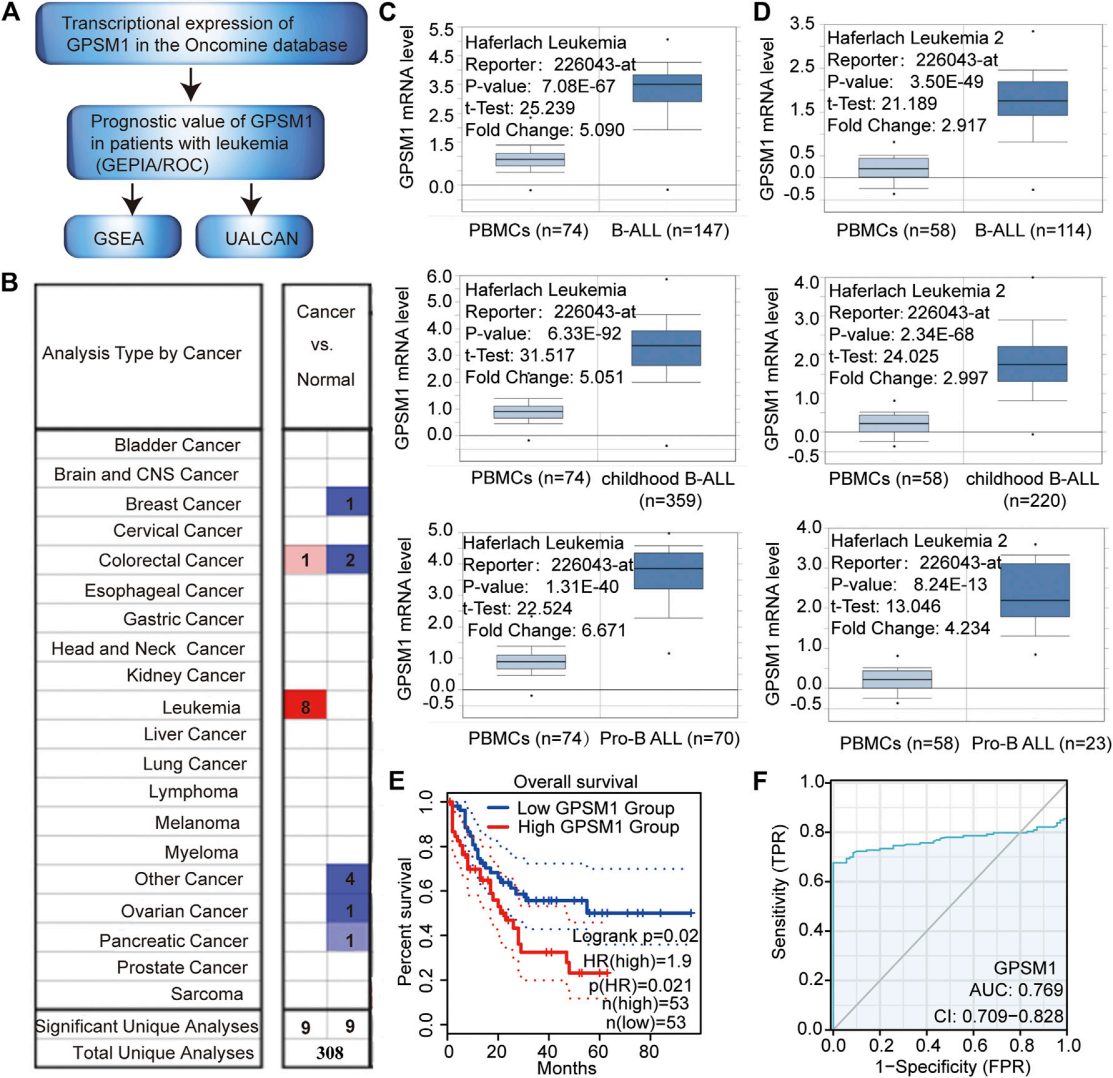
GPSM1 was highly expressed in B-ALL. **(A)** Flowchart for bioinformatic analyses. **(B)** Transcriptional expression of GPSM1 in 20 different types of cancer from the Oncomine database. Differences in transcriptional expression were compared by Student’s *t* test. The cutoff values and analysis parameters were as follows: *p* value: 0.05, fold change: 2, gene rank: 10%, data type: mRNA. **(C)** mRNA expression of GPSM1 in PBMCs **(left)** and B-ALL **(right)** (Haferlach Leukemia). **(D)** mRNA expression of GPSM1 in PBMCs **(left)** and B-ALL **(right)** (Haferlach Leukemia 2). **(E)** Kaplan-Meier curve was used for the relationship between GPSM1 expression and OS in LAML in the GEPIA, demonstrating that a high level of GPSM1 was associated with poor prognosis in LAML (*n* = 106). **(F)** ROC analysis of GPSM1 expression showing moderate discrimination power between acute myeloid leukemia patients (173) and healthy people (70).

To determine the function of GPSM1 in tumors, we explored GPSM1 through bioinformatics analysis (Figure 1A). As shown in Figure 1B, the mRNA expression of GPSM1 in 20 types of cancers was compared to that in normal tissues by means of the Oncomine database. Significantly higher mRNA expression of GPSM1 was found in B-ALL in multiple datasets. In the Haferlach Leukemia dataset, the expression levels of GPSM1 in B-ALL, childhood B-ALL and Pro-B ALL were 5.09 (*p* = 7.08E-67), 5.051 (*p* = 6.33E-92) and 6.671 (*p* = 1.31E-40) fold higher than those in PBMCs (Figure 1C). Additionally, in the Haferlach Leukemia 2 dataset, the expression levels of GPSM1 in B-ALL, childhood B-ALL and Pro-B ALL samples were 2.917 (*p* = 3.50E-49), 2.997 (*p* = 2.34E-68) and 4.234 (*p* = 8.24E-13) fold higher than those in PBMCs (Figure 1D). Taken together, our results showed that GPSM1 was highly expressed in patients with B-ALL.

To clarify the prognostic role of GPSM1 in leukemia, we used GEPIA to assess survival. The GEPIA results showed that high mRNA expression of GPSM1 was associated with poor overall survival (OS) in acute myeloid leukemia patients (HR = 1.9, *p* = 0.021) (Figure 1E). We performed ROC analysis of data from acute myeloid leukemia patients and healthy people to measure the discrimination value of the GPSM1. The AUC was 0.769, representing a moderate discrimination value for LAML (Figure 1F). These results implied that high mRNA expression of GPSM1 could significantly affect leukemia patient prognosis.

### GPSM1 Was Positively Correlated With Proliferation, GPCR Ligand Binding, Gαs Signaling and Calcium Signaling Pathways

**FIGURE 2:**
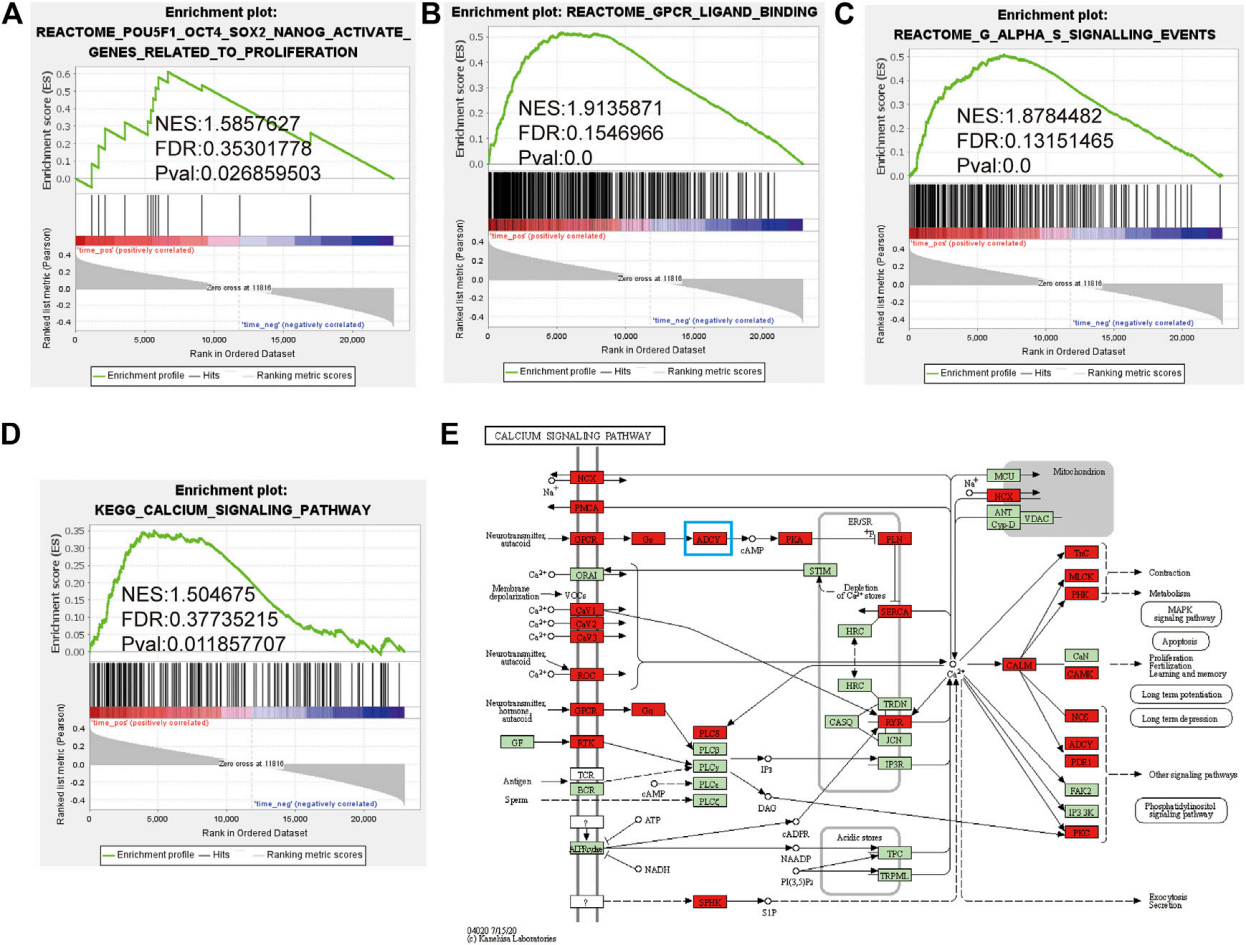
GSEA identified cell proliferation, GPCR ligand binding, Gαs signaling and calcium signaling pathways as regulatory targets of GPSM1 in the GSE87070 dataset. **(A–D)** GSEA was performed based on a Gene Expression Omnibus (GEO) dataset. NES, normalized enrichment score; FDR, false discovery rate. **(E)** Leading-edge genes in the calcium signaling pathway regulated by the GPSM1 in BCP-ALL.

GSEA is a conventional approach for identifying pathways related to gene expression. To elucidate whether GPSM1 was involved in BCP-ALL, we performed GSEA of the GSE87070 dataset. As shown in Figures 2A–D, cell proliferation, GPCR ligand binding, Gαs signaling and calcium signaling pathways were significantly associated with GPSM1 expression, which suggested that GPSM1 may play a key role in BCP-ALL. The leading-edge subset from the GSEA results can be interpreted as the group of core enriched genes (22). Interestingly, ADCY was one of the leading-edge genes in this subset and significantly contributed to the core enrichment score (Figure 2E).

### Downregulation of GPSM1 Suppressed Cell Proliferation, Induced Cell Cycle Arrest and Promoted Apoptosis in BALL-1 and Reh Cells

**FIGURE 3:**
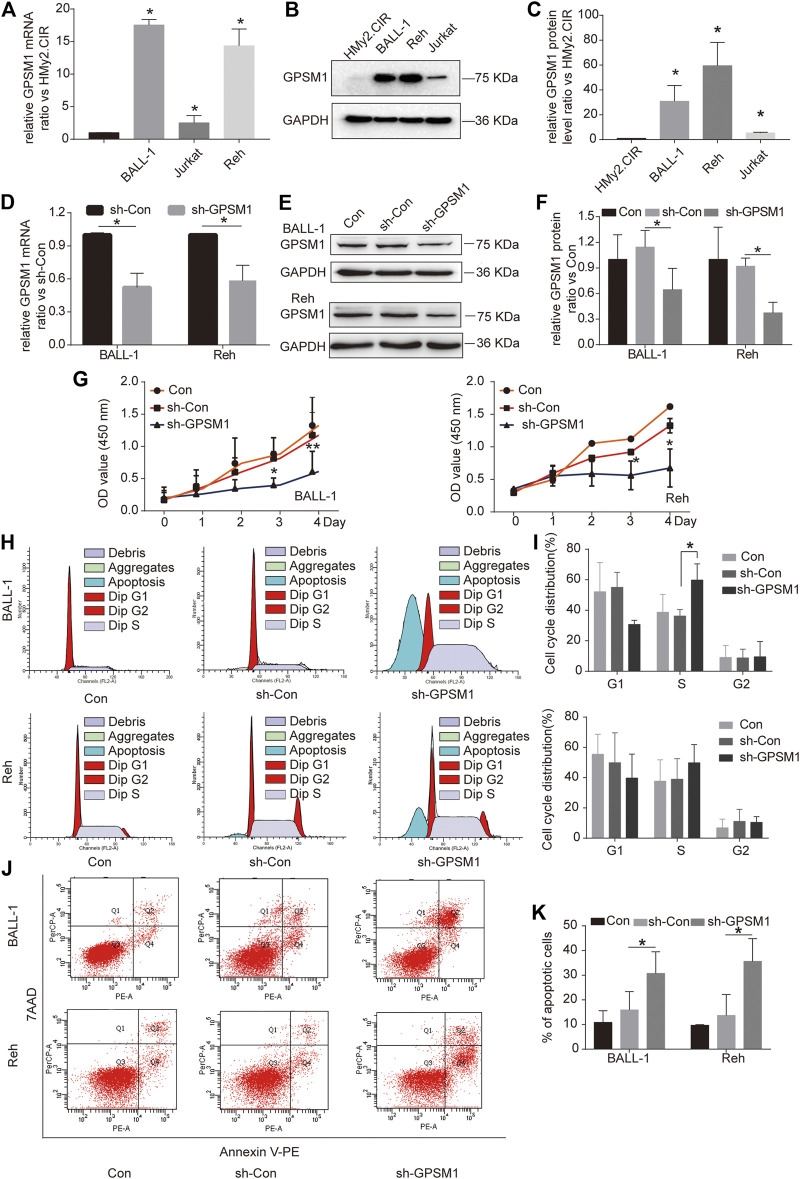
Downregulation of GPSM1 suppressed cell proliferation, induced cell cycle arrest and promoted apoptosis in BALL-1 and Reh cells. **(A)** Expression of GPSM1 mRNA in the human B lymphoblast cell line HMy2.CIR and several human leukemia cell lines (BALL-1, Jurkat and Reh). GAPDH was used as an internal reference for normalization. *p < 0.05 vs. the HMy2.CIR group. **(B)** GPSM1 protein expression in the human B lymphoblast cell lineHMy2.CIR and several human leukemia cell lines (BALL-1, Jurkat and Reh) was investigated by western blotting. **(C)** The grayscale value of the GPSM1 protein band was quantified by Tanon Image software, and GAPDH was used as an internal reference. *p < 0.05 vs. the HMy2.CIR group. **(D)** Relative mRNA expression in sh-Con- and sh-GPSM1-transfected BALL-1 and Reh cells. **(E)** GPSM1 protein expression in sh-Con- and sh-GPSM1-transfected BALL-1 and Reh cells. **(F)** The grayscale value of the GPSM1 protein band was quantified by Tanon Image software, and GAPDH was used as an internal reference. *p < 0.05, compared with the sh-Con group. **(G)** The CCK-8 assay was used to evaluate the cell proliferation ability of BALL-1 and Reh cells transfected with sh-GPSM1 and sh-Con on four consecutive days. *p < 0.05, **p < 0.01, compared with the sh-Con group. **(H,I)** The cell cycle was examined by PI staining using flow cytometry. **(J)** Cell apoptosis rates were assessed by flow cytometry after staining cells with annexin V-PE/7-AAD. Nonapoptotic cells: annexin V-PE^−^/7-AAD^−^, early apoptotic cells: annexin V-PE^+^/7-AAD^−^, late apoptotic cells: annexin V-PE^+^/7-AAD^+^. PE, phycoerythrin; 7-AAD, 7-amino-actinomycin D. **(K)** The total apoptosis rate is the sum of the early apoptosis rate and the late apoptosis rate. *p < 0.05, compared with the sh-Con group. All data are expressed as the mean ± SD.

The level of GPSM1 in ALL was assessed by RT-qPCR and western blotting. As demonstrated, the mRNA and protein levels of GPSM1 in three ALL cell lines, BALL-1, Jurkat, and Reh, were higher than those in the HMy2.CIR cell line (Figures 3A–C). BALL-1 and Reh cells, derived from B-ALL, were used for the following loss-of-function investigation. We employed adenovirus-mediated short hairpin RNA (shRNA) delivery to knockdown GPSM1 in BALL-1 and Reh cells. The mRNA interference efficiency of sh-GPSM1 in BALL-1 and Reh cells was 48 and 42%, respectively (Figure 3D). The protein interference efficiency of sh-GPSM1 in BALL-1 and Reh cells was 45 and 60%, respectively (Figures 3E,F). Compared with sh-Con-infected cells, cells infected with sh-GPSM1 exhibited significantly decreased GPSM1 mRNA and protein levels.

To dissect the function of GPSM1 in B-ALL, we assessed cell proliferation in BALL-1 and Reh cells after GPSM1 shRNA transfection. CCK-8 assays were carried out to detect cell proliferation. The results showed that knockdown of GPSM1 significantly suppressed cell proliferation in BALL-1 and Reh cells (Figure 3G). Cell cycle regulation is important for cell proliferation; therefore, the cell cycle populations of BALL-1 and Reh cells were determined by propidium iodide (PI) staining and flow cytometry. Figures 3H,I, implies that GPSM1 knockdown was able to increase the proportion of cells in S phase and reduce the proportion of cells in G1 phase. This indicated that GPSM1 knockdown blocks cell cycle progression. We further stained cells with annexin V-PE and 7-AAD to examine the apoptosis rate of BALL-1 and Reh cells. FACS was used to detect and quantify the percentage of apoptotic cells after GPSM1 viral infection. The sum of early apoptotic and late apoptotic cell percentages in the sh-GPSM1 group was remarkably elevated compared with that in the sh-Con group of BALL-1 and Reh cells (Figures 3J,K). Collectively, GPSM1 knockdown inhibited proliferation, enhanced cell apoptosis, and induced cell cycle arrest in BALL-1 and Reh cells.

### Knockdown of GPSM1 Caused ADCY6 and RAPGEF3 Downregulation in BALL-1 and Reh Cells

**FIGURE 4:**
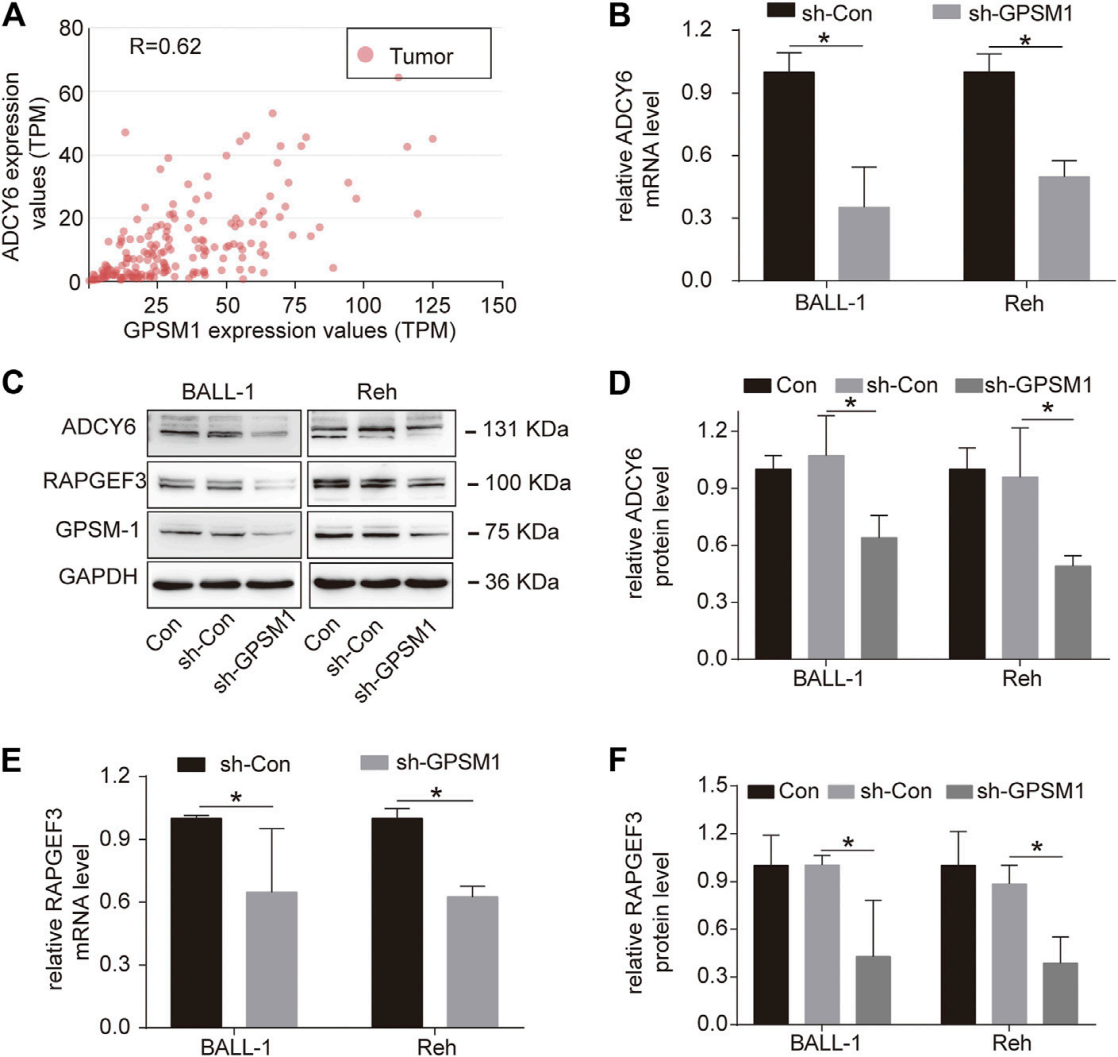
Knockdown of GPSM1 caused ADCY6 and RAPGEF3 downregulation in BALL-1 and Reh cells. **(A)** The correlation between GPSM1 and ADCY6 expression in the LAML dataset in the UALCAN. **(B, E)** The mRNA expression of ADCY6 and RAPGEF3 in BALL-1 and Reh cells after GPSM1 knockdown. **(C)** Representative western blot depicting the protein levels of ADCY6, RAPGEF3 and GPSM1 in the Con, sh-Con and sh-GPSM1 groups. GAPDH was used as the loading control. **(D,F)** Quantification of ADCY6 and RAPGEF3protein levels in the Con, sh-Con and sh-GPSM1 groups relative to GAPDH protein levels. All data are expressed as the mean ± SD. *p < 0.05, compared with the sh-Con group.

ADCY was one of the leading-edge genes in the calcium signaling pathway regulated by GPSM1 in BCP-ALL, as shown above by GSEA (Figure 2E). We used UALCAN to analyze GPSM1-related genes in the LAML dataset for acute myeloid leukemia, and the correlation analysis results showed that GPSM1 was positively correlated with ADCY6 (Figure 4A). ADCY6 was downregulated after GPSM1 knockdown in BALL-1 and Reh cells (Figures 4B–D), which corresponded to the findings in the LAML dataset analysis.

In mammals, cAMP is produced by ADCY isoforms (23). Epac1 (RAPGEF3) and Epac2 (RAPGEF4) are known to be important mediators of cAMP signaling (24). After GPSM1 knockdown, RAPGEF3 was downregulated in BALL-1 and Reh cells (Figures 4C,E,F).

### GPSM1 Knockdown Downregulated JNK Expression via RAPGEF3

**FIGURE 5:**
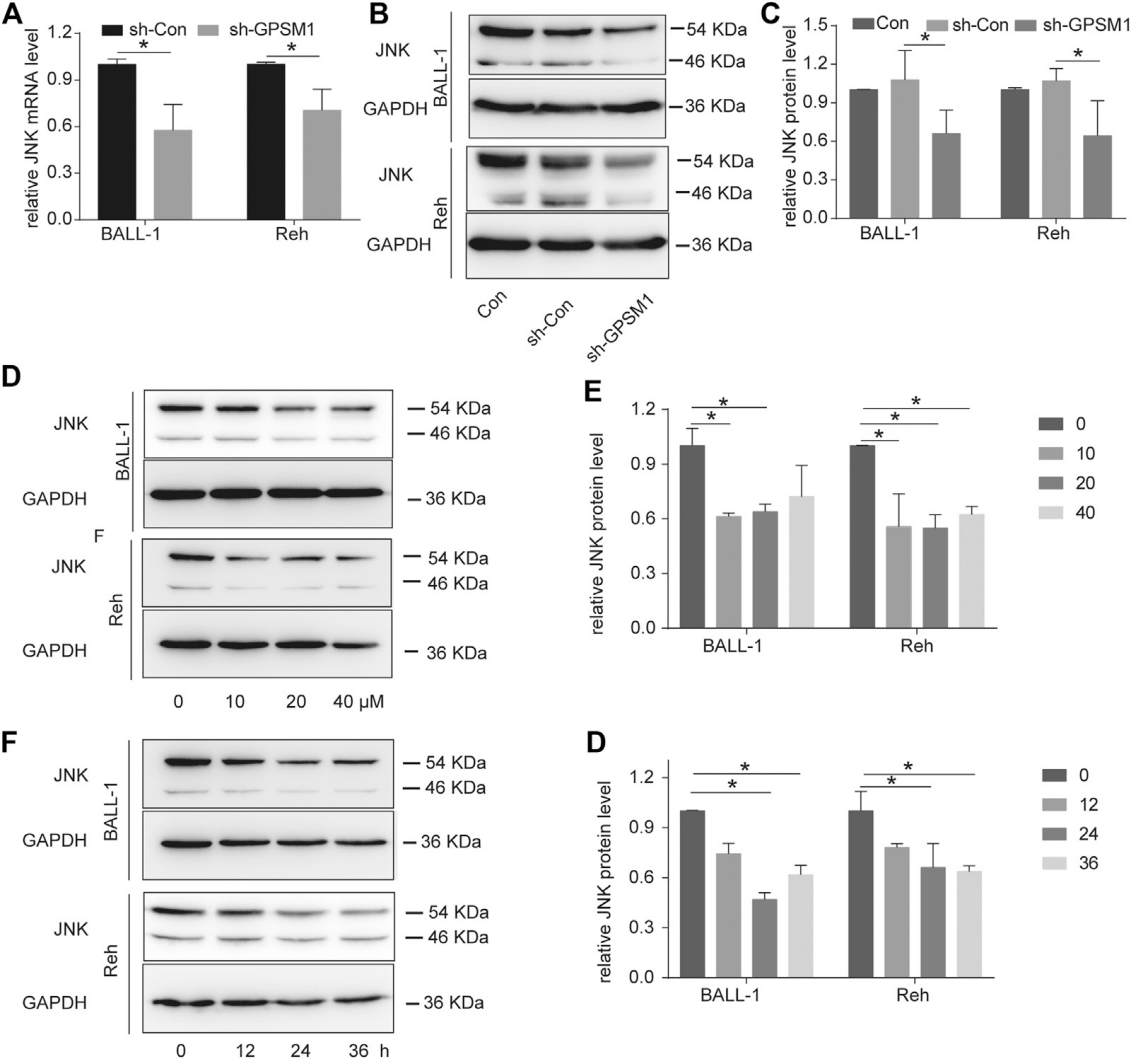
GPSM1 knockdown downregulated JNK expression via RAPGEF3. **(A)** RT-qPCR analysis of JNK expression levels in BALL-1 and Reh cells with GPSM1 knockdown. *p < 0.05, compared with the sh-Con group. **(B)** Western blot analysis of JNK expression levels after GPSM1 knockdown in BALL-1 and Reh cells. **(C)** Quantification of JNK protein levels normalized to GAPDH protein levels and plotted as fold changes relative to the levels in the Con group cells. *p < 0.05, compared with the sh-Con group. **(D)** Following a 24-h incubation with the RAPGEF3 inhibitor ESI-09 (0, 10, 20, 40 μM), BALL-1 and Reh cells were harvested for protein extraction. **(E)** The quantification of protein levels of **(D)** was performed densitometrically and normalized to GAPDH levels. *p < 0.05, compared with the 0 μM group. **(F)** Following a 0, 12, 24, 36 -h incubation with the RAPGEF3 inhibitor ESI-09 (20 μM), BALL-1 and Reh cells were harvested for protein extraction. **(G)** The quantification of protein levels in **(F)** was performed via densitometry and the target protein levels were normalized to the GAPDH levels. *p < 0.05, compared with the 0 μM group. All data were expressed as the mean ± SD.

GPSM1 binds to Gαi and leads to dissociation of Gβγ from Gαi subunits; although the precise mechanisms through which Gβγ modulates signaling have not been elucidated, Gβγ has been implicated in activation of MAPK (25). The JNK signaling pathway plays an important role in tumor cell proliferation, differentiation and survival (26). To study whether GPSM1 expression has an effect on the JNK signaling pathway, we assessed the expression of JNK in BALL-1 and Reh cells after GPSM1 knockdown. The results showed that JNK was downregulated after GPSM1 knockdown in BALL-1 and Reh cells (Figures 5A–C). To verify whether GPSM1 regulates JNK by modulating RAPGEF3, the RAPGEF3 inhibitor ESI-09 was used in further studies. The results depicted in Figures 5D–G clearly indicate ESI-09 downregulated the expression of JNK in BALL-1 and Reh cells in the dose- and time-dependent manner. This further confirmed that RAPGEF3 mediates JNK upregulation.

## Discussion

Leukemia is one of the most prevalent cancers and accounted for 3.2% of cancer-related death worldwide in 2018 (27). Several studies have shown the association of GPSM1 with the progression of multiple myeloma (28), prostate cancer (29) and esophageal squamous cell carcinoma (30), but the role of GPSM1 in the development of leukemia has not been defined. The analysis of several datasets extracted from the Oncomine database showed that the expression of GPSM1 in B-ALL is significantly higher than that in controls (Figure 1). Moreover, we observed that GPSM1 was upregulated in BALL-1, Jurkat and Reh leukemia cell lines compared to control cells (Figure 3). Additionally, GEPIA survival analysis suggested that high expression of GPSM1 was associated with poor OS in the LAML datasets (Figure 1E). The ROC curve exhibited a moderate discriminating capacity of the GPSM1 for LAML (Figure 1F). Altogether, these results suggest that GPSM1 might be a novel therapy target for B-ALL.

We performed GSEA to elucidate the potential functions of GPSM1. Interestingly, in samples with high GPSM1 expression phenotype, many genesets were significantly enriched in proliferation-related processes (Figure 2A). Accordingly, GPSM1 promotes renal tubular epithelial cell proliferation and renal tubular regeneration (31). Knockdown of GPSM1 inhibits the proliferation of renal epithelial cells (32). Moreover, polycystic kidney disease (PKD) is characterized by abnormal proliferation of epithelial cells in the kidney. GPSM1 expression is increased in the urinary exosomes of humans with PKD compared to those of healthy controls (33). These findings suggested that GPSM1 may accelerate the proliferation. Our results were also substantiated by *in vitro* experiments showing that sh-RNA-mediated knockdown inhibited the proliferation of BALL-1 and Reh cells (Figure 3G). The cell cycle regulates cell proliferation, growth and survival. Abnormal cell cycle distribution can slow the proliferation of cancer cells and reduce their survival rate (34). The results of the PI flow cytometric assay indicated that knockdown of GPSM1 promoted S-phase arrest in the BALL-1 and Reh cell line (Figures 3H,I). We speculated that GPSM1 might affect BALL-1 and Reh proliferation by regulating the cell cycle.

Cell cycle abnormalities are closely related to cell apoptosis. GPSM1 plays an important antiapoptotic role after traumatic brain injury (35). In multiple myeloma, GPSM1 overexpression is correlated with decreased apoptosis (28). Similarly, the annexin V-PE/7-AAD results suggested that the proportion of AV^+^ 7-AAD^−^ and AV^+^ 7-AAD^+^ cells increased significantly after GPSM1 knockdown, which proved that GPSM1 knockdown induced the apoptosis of BALL-1 and Reh cells. The above results indicated that GPSM1 promotes proliferation, inhibits apoptosis and induces cell cycle arrest in BALL-1 and Reh cells.

However, further pathway analyses are required to validate the changes caused by GPSM1 knockdown. According to GSEA, GPSM1 was enriched in GPCR ligand binding, Gαs signaling and calcium signaling pathways (Figures 2B–D). In addition, the leading-edge genes involved in the calcium signaling pathway included ADCY (Figure 2E). The ADCY gene family is composed of ten members (ADCY1- ADCY10). The UALCAN analysis revealed that GPSM1 was positively correlated with ADCY6 expression in the LAML dataset. Consistent with these results, knockdown of GPSM1 reduced the expression of ADCY6 in BALL-1 and Reh cells, which indicated that GPSM1 might regulate ADCY6 expression in BALL-1 and Reh cells.

Each ADCY isoform produces a common signaling molecule, cAMP. cAMP stimulates several downstream effectors, such as PKA and Epac (36). There are two isoforms of Epac, Epac1 (RAPGEF3) and Epac2 (RAPGEF4), which have similar cAMP binding affinities with PKA (24). In the present study, GPSM1 knockdown cells displayed downregulation of RAPGEF3 expression in BALL-1 and Reh cells. These observations suggested that GPSM1 could regulate ADCY6 and RAPGEF3.

**FIGURE 6:**
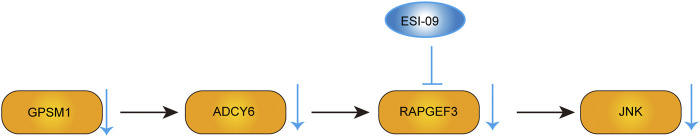
Diagram of the possible mechanism of GPSM1 in B-ALL.

Epac can directly act on the downstream signaling molecules Rap1 and JNK independently of PKA. The JNK signaling pathway plays an important role in tumor cell proliferation, differentiation and apoptosis (37). Other studies have shown that JNK activation can promote the formation of B-ALL caused by BCR/ABL genes and can promote the proliferation of T-ALL cells (38). This study showed that the expression of JNK was significantly downregulated in BALL-1 and Reh cells with GPSM1 knockdown, suggesting that GPSM1 could regulate JNK. To determine whether JNK is downstream of RAPGEF3, we used ESI-09 to inhibit RAPGEF3 in BALL-1 and Reh cells, and JNK downregulation was detected, indicating that RAPGEF3 could regulate JNK expression. Therefore, GPSM1 may participate in the pathogenesis of B-ALL through the ADCY6- RAPGEF3-JNK pathway (Figure 6).

There are some limitations to our study. First, there was a lack of clinical outcome data and *in vitro* experiments on cells obtained from patients. In addition, this study lacked *in vivo* experimental data. Experiments using animal models should also be included in any future research. Nevertheless, our study provides useful insight into the effect of GPSM1 on cell proliferation, the cell cycle, and apoptosis in the BALL-1 and Reh cell line, but future studies are still needed.

In conclusion, the current study suggests that GPSM1 expression plays a significant role in the pathogenesis of B-ALL by regulating the ADCY6- RAPGEF3-JNK pathway. Our findings provide new insights into the molecular details of the GPSM1-associated pathway in leukemia and propose potential therapeutic targets for this disease.

## Data Availability

Publicly available datasets were analyzed in this study. This data can be found here: https://www.ncbi.nlm.nih.gov/geo/query/acc.cgi?acc=GSE87070.

## References

[1] Nathwani SM Greene LM Butini S Campiani G Williams DC Samali A The pyrrolo-1,5-benzoxazepine, PBOX-15, enhances TRAIL‐induced apoptosis by upregulation of DR5 and downregulation of core cell survival proteins in acute lymphoblastic leukaemia cells. Int J Oncol (2016) 49(1):74–88. 10.3892/ijo.2016.3518 27176505 PMC4902072

[2] Gholman RR Felemban EH El Meligy OA. Dental rehabilitation of a child with acute lymphocytic leukemia: a case report. Int J Clin Pediatr dentistry (2019) 12(6):582–6. 10.5005/jp-journals-10005-1664 PMC7229377 32440080

[3] Sriram K Insel PA. G protein-coupled receptors as targets for approved drugs: how many targets and how many drugs? Mol Pharmacol (2018) 93(4):251–8. 10.1124/mol.117.111062 29298813 PMC5820538

[4] O’Hayre M Degese MS Gutkind JS. Novel insights into G protein and G protein-coupled receptor signaling in cancer. Curr Opin Cell Biol (2014) 27:126–35. 10.1016/j.ceb.2014.01.005 24508914 PMC4021379

[5] Bond J Domaschenz R Roman-Trufero M Sabbattini P Ferreiros-Vidal I Gerrard G Direct interaction of Ikaros and Foxp1 modulates expression of the G protein-coupled receptor G2A in B-lymphocytes and acute lymphoblastic leukemia. Oncotarget (2016) 7(40):65923–36. 10.18632/oncotarget.11688 27588474 PMC5323203

[6] Maiga A Lemieux S Pabst C Lavallée V-P Bouvier M Sauvageau G Transcriptome analysis reveals that G protein-coupled receptors are potential diagnostic markers or therapeutic targets in acute myeloid leukemia. Blood (2015) 126(23):3855. 10.1182/blood.V126.23.3855.3855

[7] Tyndall JD Sandilya R. GPCR agonists and antagonists in the clinic. Med Chem (2005) 1(4):405–21. 10.2174/1573406054368675 16789897

[8] Mahadevan D Choi J Cooke L Simons B Riley C Klinkhammer T Gene expression and serum cytokine profiling of low stage CLL identify WNT/PCP, flt-3L/flt-3 and CXCL9/CXCR3 as regulators of cell proliferation, survival and migration. Hum Genomics Proteomics (2009) 2009:453634. 10.4061/2009/453634 20981323 PMC2958625

[9] Nairismägi ML Tan J Lim JQ Nagarajan S Ng CC Rajasegaran V JAK-STAT and G-protein-coupled receptor signaling pathways are frequently altered in epitheliotropic intestinal T-cell lymphoma. Leukemia (2016) 30(6):1311–9. 10.1038/leu.2016.13 26854024 PMC4895162

[10] Oner SS Maher EM Gabay M Tall GG Blumer JB Lanier SM. Regulation of the G-protein regulatory-Gαi signaling complex by nonreceptor guanine nucleotide exchange factors. J Biol Chem (2013) 288(5):3003–15. 10.1074/jbc.M112.418467 23212907 PMC3561525

[11] Blumer JB Lanier SM. Activators of G protein signaling exhibit broad functionality and define a distinct core signaling triad. Mol Pharmacol (2014) 85(3):388–96. 10.1124/mol.113.090068 24302560 PMC3935153

[12] Pattingre S De Vries L Bauvy C Chantret I Cluzeaud F Ogier-Denis E The G-protein regulator AGS3 controls an early event during macroautophagy in human intestinal HT-29 cells. J Biol Chem (2003) 278(23):20995–1002. 10.1074/jbc.M300917200 12642577

[13] Sanada K Tsai LH. G protein betagamma subunits and AGS3 control spindle orientation and asymmetric cell fate of cerebral cortical progenitors. Cell (2005) 122(1):119–31. 10.1016/j.cell.2005.05.009 16009138

[14] Vural A Al-Khodor S Cheung GY Shi CS Srinivasan L McQuiston TJ Activator of G-protein signaling 3-induced lysosomal biogenesis limits macrophage intracellular bacterial infection. J Immunol (1950) 196(2):846–56. 10.4049/jimmunol.1501595 PMC4811337 26667172

[15] Oner SS Vural A Lanier SM. Translocation of activator of G-protein signaling 3 to the Golgi apparatus in response to receptor activation and its effect on the trans-Golgi network. J Biol Chem (2013) 288(33):24091–103. 10.1074/jbc.M112.444505 23770668 PMC3745352

[16] Bowers MS Hopf FW Chou JK Guillory AM Chang SJ Janak PH Nucleus accumbens AGS3 expression drives ethanol seeking through G betagamma. Proc Natl Acad Sci USA (2008) 105(34):12533–8. 10.1073/pnas.0706999105 18719114 PMC2527946

[17] Kwon M Pavlov TS Nozu K Rasmussen SA Ilatovskaya DV Lerch-Gaggl A G-protein signaling modulator 1 deficiency accelerates cystic disease in an orthologous mouse model of autosomal dominant polycystic kidney disease. Proc Natl Acad Sci USA (2012) 109(52):21462–7. 10.1073/pnas.1216830110 23236168 PMC3535663

[18] Branham-O’Connor M Robichaux WG 3rd Zhang XK Cho H Kehrl JH Lanier SM Defective chemokine signal integration in leukocytes lacking activator of G protein signaling 3 (AGS3). J Biol Chem (2014) 289(15):10738–47. 10.1074/jbc.M113.515031 24573680 PMC4036190

[19] Rhodes DR Kalyana-Sundaram S Mahavisno V Varambally R Yu J Briggs BB Oncomine 3.0: genes, pathways, and networks in a collection of 18,000 cancer gene expression profiles. Neoplasia (2007) 9(2):166–80. 10.1593/neo.07112 17356713 PMC1813932

[20] Tang Z Li C Kang B Gao G Li C Zhang Z. GEPIA: a web server for cancer and normal gene expression profiling and interactive analyses. Nucleic Acids Res (2017) 45(W1):W98–102. 10.1093/nar/gkx247 28407145 PMC5570223

[21] Robin X Turck N Hainard A Tiberti N Lisacek F Sanchez JC pROC: an open-source package for R and S+ to analyze and compare ROC curves. BMC Bioinformatics (2011) 12:77. 10.1186/1471-2105-12-77 21414208 PMC3068975

[22] Subramanian A Tamayo P Mootha VK Mukherjee S Ebert BL Gillette MA Gene set enrichment analysis: a knowledge-based approach for interpreting genome-wide expression profiles. Proc Natl Acad Sci USA (2005) 102(43):15545–50. 10.1073/pnas.0506580102 16199517 PMC1239896

[23] Halls ML Cooper DMF. Adenylyl cyclase signalling complexes–pharmacological challenges and opportunities. Pharmacol Ther (2017) 172:171–80. 10.1016/j.pharmthera.2017.01.001 28132906

[24] Bos JL. Epac proteins: multi-purpose cAMP targets. Trends Biochem Sci (2006) 31(12):680–6. 10.1016/j.tibs.2006.10.002 17084085

[25] Schwindinger WF Robishaw JD. Heterotrimeric G-protein betagamma-dimers in growth and differentiation. Oncogene (2001) 20(13):1653–60. 10.1038/sj.onc.1204181 11313913

[26] Hammouda MB Ford AE Liu Y Zhang JY. The JNK signaling pathway in inflammatory skin disorders and cancer. Cells (2020) 9(4). 10.3390/cells9040857 PMC7226813 32252279

[27] Bray F Ferlay J Soerjomataram I Siegel RL Torre LA Jemal A Global cancer statistics 2018: GLOBOCAN estimates of incidence and mortality worldwide for 36 cancers in 185 countries. CA Cancer J Clin (2018) 68(6):394–424. 10.3322/caac.21492 30207593

[28] Shao S Huang X Wang Y He S Xu X Zhu X A role for activator of G-protein signaling 3 (AGS3) in multiple myeloma. Int J Hematol (2014) 99(1):57–68. 10.1007/s12185-013-1484-8 24307516

[29] Adekoya TO Smith N Aladeniyi T Blumer JB Chen XL Richardson RM. Activator of G protein signaling 3 modulates prostate tumor development and progression. Carcinogenesis (2019) 40(12):1504–13. 10.1093/carcin/bgz076 31215992 PMC7346714

[30] Shi H Ren H Yang X Zhu H Yao L Hang Q Overexpression of activator of G-protein signaling 3 decreases the proliferation of esophageal squamous cell carcinoma. Pathol Res Pract (2015) 211(6):449–55. 10.1016/j.prp.2014.12.016 25812748

[31] Regner KR Nozu K Lanier SM Blumer JB Avner ED Sweeney WE Jr Loss of activator of G-protein signaling 3 impairs renal tubular regeneration following acute kidney injury in rodents. FASEB J (2011) 25(6):1844–55. 10.1096/fj.10-169797 21343176 PMC3101034

[32] Nadella R Blumer JB Jia G Kwon M Akbulut T Qian F Activator of G protein signaling 3 promotes epithelial cell proliferation in PKD. J Am Soc Nephrol (2010) 21(8):1275–80. 10.1681/ASN.2009121224 20488951 PMC2938587

[33] Keri KC Regner KR Dall AT Park F. Urinary exosomal expression of activator of G protein signaling 3 in polycystic kidney disease. BMC Res Notes (2018) 11(1):359. 10.1186/s13104-018-3467-6 29880041 PMC5992714

[34] Hirsch GE Parisi MM Martins LA Andrade CM Barbé-Tuana FM Guma FT. γ-oryzanol reduces caveolin-1 and PCGEM1 expression, markers of aggressiveness in prostate cancer cell lines. Prostate (2015) 75(8):783–97. 10.1002/pros.22960 25619388

[35] Wei W Qi L Feihui Z Zhihua Y Yunfeng W Ting L Increased expression of AGS3 in rat brain cortex after traumatic brain injury. J Neurosci Res (2013) 91(5):726–36. 10.1002/jnr.23195 23404409

[36] Patra C Foster K Corley JE Dimri M Brady MF. Biochemistry, cAMP. In: StatPearls [Internet]. Treasure Island (FL): StatPearls Publishing (2020). 30571052

[37] La Marca JE Richardson HE Two-faced: roles of JNK signalling during tumourigenesis in the Drosophila model. Front Cell Dev Biol (2020) 8:42. 10.3389/fcell.2020.00042 32117973 PMC7012784

[38] Leventaki V Drakos E Karanikou M Psatha K Lin P Schlette E c-JUN N-terminal kinase (JNK) is activated and contributes to tumor cell proliferation in classical Hodgkin lymphoma. Hum Pathol (2014) 45(3):565–72. 10.1016/j.humpath.2013.10.024 24457077

